# Supplementary material for: Large-Emitting-Area Quantum Dot Light-Emitting Diodes Fabricated by an All-Solution Process
Source: Int J Mol Sci. 2023 Sep 20;24(18):14350. doi: 10.3390/ijms241814350 (PMC10532160; doi:10.3390/ijms241814350)
Supplement: Supplementary file 1 [file ijms-24-14350-s001.zip › ijms-2593400-supplementary.pdf]

Supplementary Document:

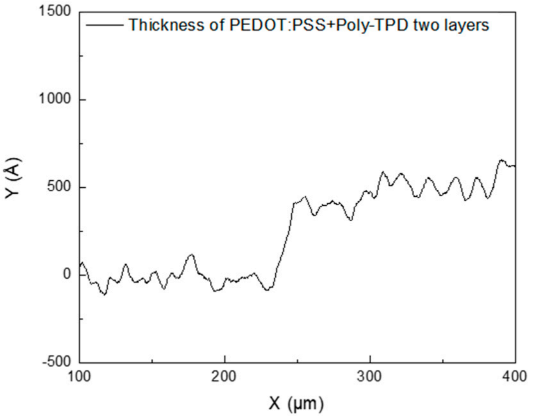

Figure S1. Total thickness of PEDOT: PSS and poly-TPD two layers

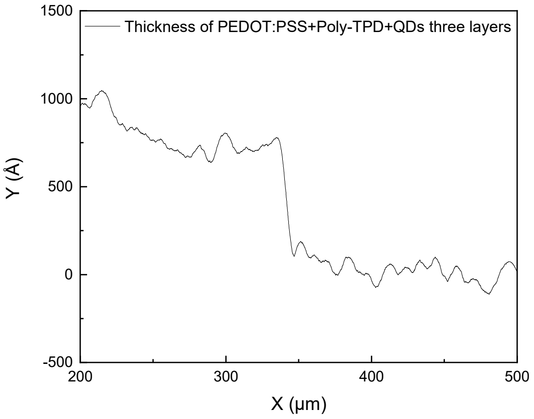

Figure S2. Total thickness of PEDOT: PSS, poly-TPD and QDs three layers

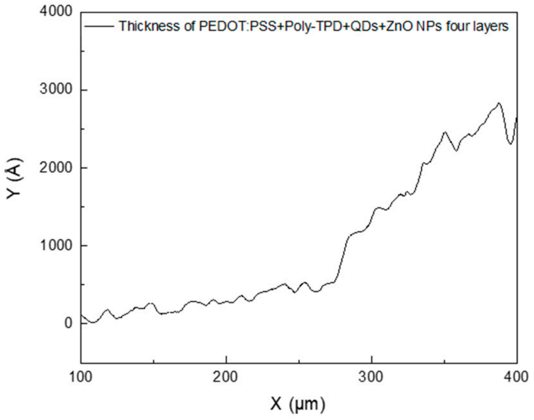

Figure S3. Total thickness of PEDOT: PSS, ply-TPD, QDs and ZnO nanoparticles four layers

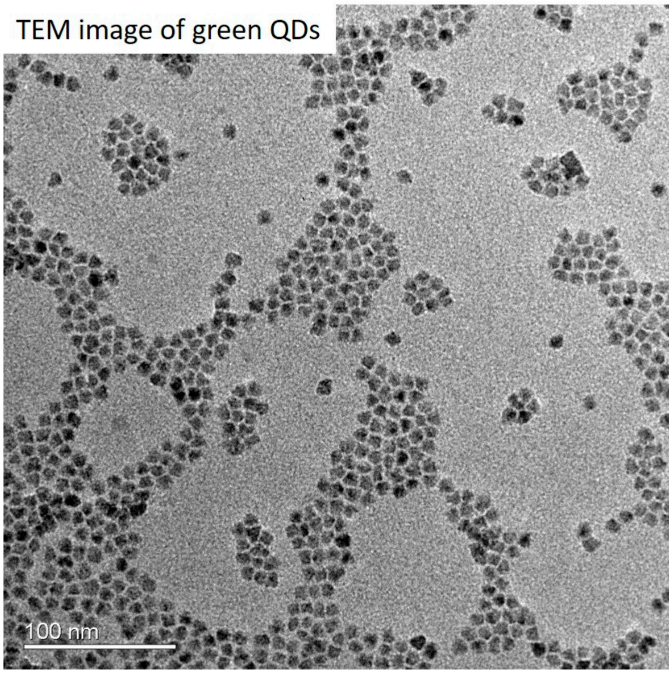

Figure S4. TEM image of green QDs.

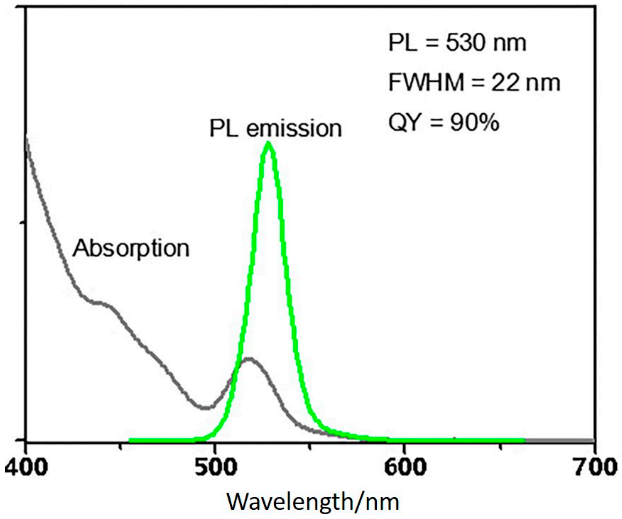

Figure S5. Normalized absorption and fluorescence spectra of green QDs.
